# DNA methyltransferase-1 in acute myeloid leukaemia: beyond the maintenance of DNA methylation

**DOI:** 10.1080/07853890.2022.2099578

**Published:** 2022-07-15

**Authors:** Mengyuan Li, Donghua Zhang

**Affiliations:** Department of Hematology, Tongji Hospital, Tongji Medical College, Huazhong University of Science and Technology, Wuhan, P.R. China

**Keywords:** Acute myeloid leukaemia, DNA methyltransferase-1, drug resistance, targeted therapy, epigenetics

## Abstract

DNA methylation is considered an essential epigenetic event during leukaemogenesis and the emergence of drug resistance, which is primarily regulated by DNA methyltransferases. DNA methyltransferase-1 (DNMT1) is one of the members of DNA methyltransferases, in charge of maintaining established methylation. Recently, DNMT1 is shown to promote malignant events of cancers through the epigenetic and non-epigenetic processes. Increasing studies in solid tumours have identified DNMT1 as a therapeutic target and a regulator of therapy resistance; however, it is unclear whether DNMT1 is a critical regulator in acute myeloid leukaemia (AML) and how it works. In this review, we summarized the recent understanding of DNMT1 in normal haematopoiesis and AML and discussed the possible functions of DNMT1 in promoting the development of AML and predicting the sensitivity of hypomethylation agents to better understand the relationship between DNMT1 and AML and to look for new hope to treat AML patients.

**Key messages**
- The function of DNA methyltransferase-1 in acute myeloid leukaemia.
- DNA methyltransferase-1 predicts the sensitivity of drug and involves the emergence of drug resistance.

## 1. Introduction

Acute myeloid leukaemia (AML) is a heterogeneous disease that is characterized by a clonal disorder of haemopoietic progenitor cells. Generally, the development of AML involves different cytogenetic abnormalities, in which the long arm of chromosome 11 (11q) and balanced translocations between chromosomes 15 and 17, t (15;17), are the most common cytogenetic abnormalities [1]. In addition, other cytogenetics and the mutational status of various genes have also appeared in AML patients [2]. Currently, the “7 + 3” chemotherapy regimen (continuous injections of cytarabine for 7 days followed by 3 days of anthracycline) is the primary treatment choice for AML patients [2]. Targeting definite molecules, such as FLT3, IDH1/2 and BCL-2, has improved the efficiency of therapy [3]. However, only 60%∼80% of patients are sensitive to standard chemotherapy [4]. In addition, patients over 60 years of age often cannot tolerate intensive chemotherapy and have a higher rate of poor-risk cytogenetics or genetic mutations, while targeted therapies can only target specific populations and are prone to drug resistance [1,5]. Therefore, current therapies cannot meet the treatment needs of some AML patients. Recent studies suggested that the genetic heterogeneity of leukaemia and haematopoietic stem cell transformation were the source of leukaemia relapse and treatment resistance [6], whereas the specific mechanism is still unknown, which forces us to explore deeper mechanisms to overcome clinical problems.

Aberrant methylation of DNA has been observed frequently in tumours and is involved in inducing genomic instability to regulate the expression of key genes to promote the malignant progression of tumours [7]. This is also common in AML and has been identified as an important mechanism for hastening disease progression and drug resistance. Thus, targeting aberrant DNA methylation is a promising therapy for AML [8,9], and hypomethylation agents have been approved for the clinic, in combination with chemotherapy or targeted agents to treat patients with newly diagnosed or refractory relapsed AML [10]. DNA methylation is primarily regulated by DNA methyltransferases (DNMTs), which catalyse the process of DNA methylation by transferring a methyl group onto the fifth carbon position of cytosine. DNMTs include three major members, termed DNMT1, DNMT3A, and DNMT3B, in which DNMT3A and DNMT3B are mainly responsible for methylation establishment while DNMT1 maintains methylation during DNA replication [11,12]. Notably, DNMTs have been reported to play a significant role during haematopoietic development, and are closely associated with the development of haematological malignancies [13].

Unlike DNMT3A and DNMT3B, DNMT1 preferentially binds to hemi-methylated cytosine-guanine (CpG) dinucleotides rather than unmethylated counterparts. In many solid tumours, overexpressed DNMT1 has been proven to induce cancer growth or proliferation and drug resistance. For example, in pancreatic ductal adenocarcinomas (PDACs), DNMT1 induces cell cycle progression and proliferation of PDAC cells and suppresses their differentiation. Noteworthily, DNMT1 promotes the self-renewal capacity of PDAC cancer stem cells which are always resistant to conventional chemotherapy and radiotherapy [14]. Similar to the results above, self-renewal of liver cancer stem cells [15], breast cancer stem cells [16] and leukaemia stem cells [17] are regulated by DNMT1.

Nevertheless, the specific mechanism of DNMT1 works in AML has yet to be determined. Consequently, a better understanding of the molecular events of DNMT1 contributing to drug resistance would facilitate the development of strategies for sustained remission. Here, we will elucidate DNMT1 from four aspects: structure, function, drug resistance related to overexpressed DNMT1, and targeted therapy. Highlighting the significance of DNMT1 in AML is very essential to explore more in-depth mechanisms of drug resistance and more therapeutic strategies.

## 2. The structure and function of DNMT1

**Figure 1.**
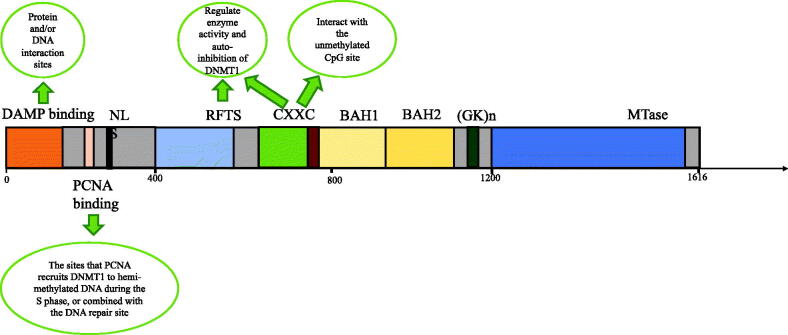
The structure of DNA methyltransferase-1.

Different from the protein structure of DNMT1 in bacteria, DNMT1 of its human counterpart is a multidomain protein, not only containing the C-terminal methyltransferase (MTase) domain but also adding a large regulatory region [18]. The DNMT1 protein comprises 1620 amino acids, with an N-terminal regulatory region covering two-thirds of the sequence and a C-terminal MTase domain, which are separated by a highly conserved (GK)n repeat [19]. The regulatory region starts with an ∼300 amino acid-long N-terminal domain (NTD) harbouring a variety of protein and/or DNA interaction sites, followed by a replication foci-targeting sequence (RFTS) domain, a CXXC zinc finger domain interacting with the unmethylated CpG site and a pair of Bromo-adjacent-homology (BAH) domains (Figure 1) [11]. It is crucial to note that these domains are involved in various functions in the activity of DNMT1. For instance, proliferating cell nuclear antigen (PCNA) recruits DNMT1 to hemi-methylated DNA sites during the S phase or the DNA repair sites by the N-terminal domain that serves as a platform for the interaction between DNMT1 and proteins or DNA, maintaining DNA methylation at the replication fork [20,21]. Furthermore, DNMT1 can interact with methyl-CpG binding proteins like UHRF1 with a ubiquitin-like with plant homeodomain (PHD) and ring finger domain1 to maintain the methylation of mammalian CG DNA [22]. By recruiting DNMT1 to hemi-methylated DNA, UHRF1 facilitates the maintenance of DNA methylation. Ubiquitin-specific protease 7 (USP7), a deubiquitinase, has strong interaction with DNMT1, which has been shown to maintain the stability of the DNMT1-UHRF1 complex and suppress excessive DNA methylation [23,24]. Through regulating the location of DNMT1, the RFTS domain and CXXC zinc finger domain mediate enzyme activity and autoinhibition of DNMT1 [25–27]. In addition, DNMT1 has an enzymatic preference of 30-40 fold for the hemi-methylated CpG site *in vitro* and provides an epigenetic mark contributing to the function of DNMT1 in replication-dependent DNA methylation [28].

As a DNA methyltransferase, DNMT1 is prone to maintain the DNA methylation pattern and de novo methylation [29,30]. Additionally, time witnessed the great advancement of studies related to DNMT1, and certain molecules interacting with DNMT1 have been unclosed.

The study, published in 2008, showed that lymphoid-specific helicase (LSH) related to the SNF2 family of chromatin-remodelling ATPases facilitated transcriptional repression by cooperating with DNMTs (DNMT1 and DNMT3B), which recruited the histone deacetylases (HDACs) [31]. Additionally, the LSH protein is a key regulator of epigenetic modifications by linking with UHRF1 to indirectly regulate DNMT1 [32,33]. In mammalian cells, p53 protein as a tumour suppressor may induce cell cycle arrest and apoptosis in the case of some stimulations of cellular stresses [34]. Survivin, a member of the inhibitor of apoptosis (IAP) family, is downregulated by activation of p53 [35]. DNMT1 binding to p53 *in vivo* is localized on the survivin promoter, which results in hypermethylation of the survivin promoter, thus contributing to the repression of the gene expression [36]. It has been reported that EZH2 and DNMT1 interact in silencing exact target genes by epigenetic processes [37]. Later, as it turns out, DNMT1 holds a seat in mediating DNA repair, cell cycle control, and regulation of apoptosis and embryonic development. Knocking out DNMT1 in mice is embryonic lethal, as it violates normal DNA methylation patterns, which are critical for embryonic development [38]. To elucidate the relationship between DNMT1 and genomic instability, Loughery et al. knocked down DNMT1 with a short hairpin RNA construct in normal human fibroblasts (hTERT-1604). The DNMT1 knockdown cells carried mismatch repair (MMR) deficiency, leading to a subsequent decrease in cell viability, which confirmed that DNMT1 played a role in the response to DNA repair [39].

More importantly, accumulating evidence indicates that DNMT1 acts as an oncogene in several cancers [40,41]. A study reported that DNMT1 is involved in the promotion of hepatocellular carcinoma, while this process has been proven to be inhibited by miR-185 by targeting the DNMT1/PTEN/AKT pathway [42]. In human osteosarcoma cells, the DNMT1/miR-34a axis has been found to play a major role in the maintenance of the osteosarcoma stem cells, leading to the development of tumours [43]. Another study reported that Isovitexin could suppress the features of human osteosarcoma stem-like cells by interfering with the DNMT1/miR-34a/Bcl-2 axis, which provides a clearer mechanism of apoptotic cell death induced by Isovitexin and is crucial to explore the causes of chemoresistance [44]. In bladder cancer, CpG site methylation of the PTEN promoter dependent on DNMT1 contributes to the poor expression of PTEN, which is regarded as a suppressor of cancers [45]. In triple-negative breast cancer (TNBC), the most aggressive subtype of breast cancer, the long noncoding RNA LINC00152 has been shown to promote TNBC tumourigenesis by activating DNMT1 [46]. Further research has been performed and found that poor breast cancer survival was associated with the overexpression of DNMT1, which is commonly observed in TNBC [47].

## 3. The function of DNMT1 in normal haematopoiesis

The past few decades have witnessed advances in epigenetic research in haematopoiesis and embryogenesis, and mounting evidence suggests that epigenetic mechanisms, especially DNA methylation, that control transcriptional programs, are essential in haematopoiesis. As reported, DNMT1 is required for haematopoietic stem cells (HSCs) to survive and develop. HSCs tend to express higher levels of DNMT1, and the study revealed an obvious decrease in HSCs and showed a near-complete absence of phenotypic HSCs and early progenitors in knockdown DNMT1 mice. Moreover, the loss of DNMT1 completely disrupted the differentiation potential of haematopoietic stem and progenitor cells. The decline in DNMT1 activity, however, cannot suppress key myeloid-erythroid regulators in HSCs from mice and thus cannot prevent the differentiation into myeloid-erythroid, but not into lymphoid progeny [48]. Since then, Trowbridge JJ et al. established a conditional knockout model, demonstrating that the loss of DNMT1 contributed to the absence of haematopoiesis and verified that DNMT1 was required for the HSCs self-renewal *in vivo* [49]. Furthermore, defects of HSCs driven by DNMT1 deficiency are C/EBPA dependent, which has been confirmed in zebrafish [50]. Maintaining normal DNMT1 function is significant for the regulation of the C/EBPA, whereas intact C/EBPA function is necessary for HSCs proliferation blockade.

## 4. The function of DNMT1 in acute myeloid leukaemia (AML)

### 4.1. DNMT1 regulates DNA methylation in AML

In the human genome, 70–90% of CpG sites are normally methylated [51]. However, the CpG sites in CpG islands concentrated with CpGs are often unmethylated in normal somatic cells [52]. It is well established that normal epigenetic processes are disrupted during the initiation and progression of tumourigenesis, including global changes to normal DNA methylation patterns. This is a vital epigenetic characteristic of cancers with malignant biotic processes, which show overall genome-wide hypomethylation and specific regional DNA hypermethylation of CpG island promoters [53]. Furthermore, de novo DNA methylation of promoter CpG islands is a frequent alteration in cancer, resulting in transcriptional silencing of dozens to hundreds of genes per tumour [54]. A typical example is the CDKN2A gene whose promoter is methylated, leading to its silencing and functional deficiency. In research related to follicular lymphoma, the authors showed that the deletion or methylation of CDKN2A was associated with poor overall survival (OS) [55]. This result demonstrated that the silencing of tumour suppressor genes induced by DNA methylation played a crucial role in aggressive disease. Similarly, abnormal DNA methylation patterns have been considered the important regulatory mechanism in the pathogenesis of leukaemias [56]. Due to the genetic heterogeneity within the precursor cells of AML, the generation of AML is regarded as the result of multiple mutation accumulation [2,57]. Unfortunately, it is not exactly clear whether DNA methylation is the cause or result of AML. A model proposed by Max Jan et al determined that serial mutations and/or epigenetic events must accumulate in self-renewing haematopoietic stem cells (HSCs) unless a mutation confers self-renewal ability on a downstream cell. They identified preleukaemic mutations in residual HSCs from AML with FLT3-ITD mutations [58]. Although the results cannot represent the evolutionary path of leukaemic cells in all cases of de novo AML, they provide a mechanism that may explain how epigenetic modifications, including DNA methylation work during the development of AML.

It has been reported that the loss of DNMT1 results in global and gene-specific demethylation and re-expression of tumour-suppressor genes in human cancer cells, which demonstrates that DNMT1 is necessary to maintain global methylation and aberrant CpG island methylation in human cancer cells [41]. Not only does the overexpression of DNMT1 exist in solid tumours and promote the occurrence of adverse events, but a similar phenomenon has also been observed in leukaemia [59,60]. Shinichi Mizuno et al. analysed DNMTs expression levels of 33 AML cases by competitive PCR and found that DNMT1 was expressed at a high level in most AML cases. Overexpression of DNMT1 could be associated with the development and relapse of AML triggered by hypermethylation of tumour suppressor genes [61]. The CpG islands of the EBF3, TMEM176A, DFNA5, and SOCS1 genes are observed at an increasing methylation level in cancers, which often causes the dysfunctions of these genes and the decrease of gene expression [62–66]. MiRNA-370, as a tumour suppressor, is downregulated due to aberrant CpG island methylation in AML, which subsequently results in the upregulation of FOXM1, a tumour-promoting factor. Consequently, these events contribute to the occurrence and progression of AML [67]. Notably, DNMT1 is bound with the deacetylase enzyme 1 (HDAC1), which could be necessary for the process of DNA methylation induced by DNMT1 [68]. Similar to the HDAC1/DNMT1 complex, the histone methyltransferase EZH2, a significant molecule that recruits and binds DNMT1 to regulate DNA methylation, forms a complex with DNMT1 in AML, which could be a target to exert antileukaemia activity [69]. What’s more, LINC00173 has been found to interact with DNMT1 in AML, affecting the level of self-promoter methylation and self-expression. In return, LINC00173 inhibits the expression of DNMT1, blocking tumour cell proliferation and enhancing the sensitivity to chemotherapy [70]. A transcription factor named Sp1 is usually observed to form a complex with NF-kB and then in combination with the promoter of DNMT1 to promote its expression, followed by hypermethylation of specific gene promoters and silenced gene transcription [71]. Actually, a link between DNMT1 and NF-kB has been reported previously and could be regulated directly by nucleolin resulting in the disruption of AML leukaemogenesis [72].

### 4.2. DNMT1 works in AML by the non-epigenetic process

Above we discussed how DNMT1 participates in the different biological processes of AML by regulating DNA methylation; however, DNMT1 also interacts with some crucial regulating factors, which belongs to non-epigenetic processes. Fatty acid-binding protein 4 (FABP4) has been reported to promote AML aggressiveness through enhanced DNMT1-dependent DNA methylation, FABP4 regulates DNMT1 expression through the IL-6/STAT3 axis, whereas DNMT1 controls FABP4 through VEGF signal pathway, which constitutes a feedback loop, in turn, affecting the process of leukaemia [73]. Currently, most studies focus on aberrant methylation regulated by DNMT1, while this study provided a new understanding of the function of DNMT1 in AML, particularly from a metabolic standpoint. Furthermore, USP7 was identified as an interaction partner of DNMT1 that could regulate its stability and activity by the connection with the TS domain of DNMT1 and domain 3 of USP7, then affecting the survival outcomes of the haematological malignancies [24,74]. AML1-ETO AML derives from the translocation t (8;21) (q22; q22), creating a novel chimeric gene, RUNX1/MTG8, AML patients with this genetic abnormality usually achieve a relatively favourable prognosis [75]. It has been reported that a physical and functional interaction between endogenous RUNX1/MTG8 and endogenous DNMT1 exists in Kasumi-1 cells, and RUNX1/MTG8 modulates its targeted genes by recruiting DNMT1 to their promoters [76]. Based on these results of the study, it is not difficult to find that DNMT1 is an important molecule in AML1-ETO AML, whereas a specific interacting site has been far from clear. Moreover, the stem cell factor spalt-like transcription factor 4 (SALL4) is a key molecule in normal haematopoiesis and also in leukaemogenesis, which blocks self-repression by recruiting DNMT1 to its promoter, which affects its function in AML [77,78].

### 4.3. The function of DNMT1 in leukaemic stem cells (LSCs)

According to research findings, DNMT1 sustains a higher level of expression in leukaemic stem cells (LSCs) compared with chemo-sensitive leukaemia cells, which could maintain the self-renewal of LSCs and regulate the biological functions of LSCs [17]. As mentioned above, SALL4 holds self-expression by recruiting DNMT1 and influences epigenetics, thus playing an important role in maintaining LSCs self-renewal [78,79]. MLL-AF9 AML caused by the MLL fusion gene is an important type of AML, conferring a worse prognosis, and the mice with MLL-AF9 AML have been considered a great model to study LSCs [80]. A previous study demonstrated that hypomethylation induced by the loss of DNMT1 impaired the self-renewal of MLL-AF9 LSCs [48]. Likewise, conditional knockout of DNMT1 damages the methylation patterns and survival of LSCs. MLL-AF9-induced AML depends on DNMT1 in transformation giving rise to leukaemia, maintenance of established leukaemia, and re-establishment of leukaemia by transplanting L-GMPs [17].

**Figure 2.**
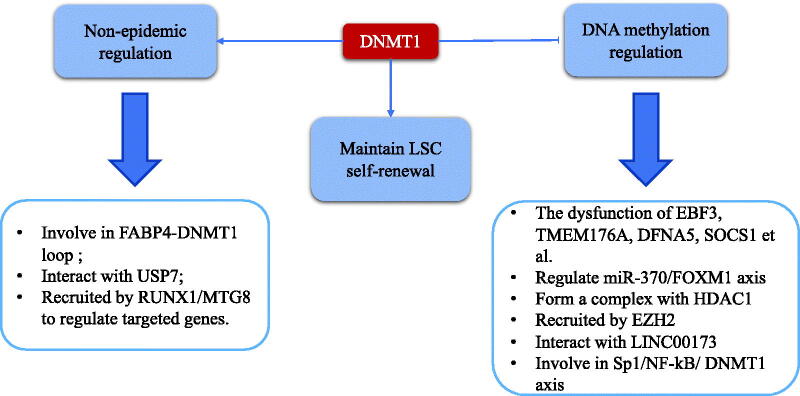
The function of DNA methyltransferase-1 in acute myeloid leukaemia.

Overall, DNMT1 plays a crucial role in the different stages of AML not only by inducing aberrant methylation profile but also by non-methylation programs, which could promote the development of the disease and mediate resistance to AML cell death (Figure 2).

## 5. DNMT1 is considered a regulator of drug resistance and a marker predicting the sensitivity of demethylation drugs in AML

Currently, DNMT inhibitors (DNMTi) are considered non-intensive therapy applied in myelodysplastic syndromes (MDS) and AML [81]. Accumulating studies have focussed on decitabine (a DNMTi), which has been shown to target DNMT1 and has been approved for the treatment of MDS and AML by the FDA. The working model of the drug is processed by pyrimidine metabolism into a deoxycytidine triphosphate (dCTP) analog, Aza-dCTP, followed by degradation of the key epigenetic regulator DNMT1 from dividing cells, then inducing demethylation to activate silenced genes [82]. Cytarabine is the most common chemotherapy, forming the standard chemotherapy regimen for AML patients. Since the recent combination of decitabine with cytarabine has acquired notable effects, many clinical trials have concentrated on combining regulating epigenetic drugs with cytarabine to treat refractory and high-risk AML [83,84]. A previous study also acquired the same synergistic action in AML cell lines by combining decitabine with cytarabine. A low level of DNA methylation was induced by DNMT1 deficiency resulting from decitabine, which increased the sensitivity of AML cell lines to cytarabine [85]. The combination of decitabine with cladribine, clofarabine, fludarabine, and busulfan decreased the expression level of DNMT1, which increased the expression of genes silenced by epigenetic modifications and the cytotoxicity [86]. However, it remains a mystery how decitabine increases the sensitivity to cytarabine and whether DNMT1 functions during the generation of cytarabine resistance. Cancer stem cells are difficult to clear by chemotherapy, which may lead to chemotherapy resistance, and the mechanism has also been reported in AML [87]. We speculate that DNMT1 may mediate the chemoresistance by maintaining LSCs as described above. Furthermore, DNMT1 has also been found to be involved in the resistance of hypomethylation agents, which may result from incompletely depletion of DNMT1 after treatment with decitabine or 5-azacytidine [88]. Despite leading to the reactivation of key regulatory genes by reducing gene promoter methylation levels, inducing the DNA damage response is considered another antitumour effect of decitabine [89]. However, Stela S Palii et al. revealed that DNMT1 colocalized with γ-H2AX foci, which mediated its accumulation to the sites of DNA damage, and a strong interaction between DNMT1 and checkpoint kinase (CHK1) promoted the response to DNA damage [90], while as a regulator of DNA damage repair, DNMT1 may interfere with the effect of decitabine on DNA damage, causing the generation of resistance. Therefore, to address the poor response of patients to treatment in the clinic, exploring the deeper mechanism of DNMT1 mediating chemoresistance and the resistance of hypomethylation agents is necessary.

It has been reported that miRNAs which are non-coding, single-stranded RNA molecules containing 18–22 nucleotides play a crucial role in normal haematopoiesis [91]. In AML, it is perhaps unsurprising that the expression of most miRNAs is deregulated. More importantly, the lower expressed miRNAs lost the function of controlling the expression of key regulators, which resulted in the disorders of haematopoiesis and the process of AML, especially the loss of several tumour-suppressive genes [67,92]. Multiple pieces of evidence have shown that the miRNAs-DNMT1 axis is involved in drug resistance. According to a study of 5-Aza-dC treatment for pancreatic cancer, the low levels of DNMT1 expression increased the sensitivity to 5-Aza-dC [93]. Francoise Solly et al. reported that DNMT1 was overexpressed in azacitidine-resistant cells. Interestingly, DNMT1 is post-transcriptionally regulated by miRNAs which are usually found to be deregulated in AML [94]. Collectively, it is insightful to explore the role that the miRNAs-DNMT1 axis plays in drug resistance, although the study focussed on azacitidine-resistant AML cells only.

Recently, the antitumour immune response is regarded as a hotspot at present treatment in AML, and it is promising to eradicate chemoresistance and replace traditional chemotherapy with serious toxicity by suppressing the immune system [95]. Intriguingly, antitumour immunity seems to be modulated by epigenetics [95]. The expression of tumour-specific antigens is associated with DNA methylation, according to the increase of NY-ESO-1 and other cancer/testis antigen expression in myeloid leukaemia cells after treatment with decitabine [96]. In addition, the overexpression of immune checkpoints and the loss of anti-immune checkpoint molecules are regarded as significant mechanisms resulting in immune escape of tumours, thus cancer checkpoint inhibitors, such as anti-PD1, anti-CTLA4, and anti-PDL1 are widely used in cancer treatments [97,98]. However, approximately 40%–65% of melanoma patients who accept anti-PD1-based therapy respond poorly, which shows that the appearance of anti-PD1 or anti-PDL1 resistance makes it difficult to target these immune checkpoints to treat patients [99]. In addition, decitabine has been shown to improve the sensitivity of anti-PD1/PDL1 in AML by regulating DNA methylation, in which DNMT1 could be a crucial regulator [100,101]. Indeed, small cell lung cancer (SCLC) patients characterized by cisplatin resistance express higher levels of PD1 and PDL1. Mechanistically, the upregulation of PD1 and PDL1 is contributed by the overexpression of DNMT1, which revealed that overexpressed DNMT1 promoted the occurrence of antitumour immune therapy resistance [102]. Similarly, the expression of PDL1 controlled by DNMT1 significantly influenced the response to chemotherapy in colorectal cancer [103]. Unfortunately, insufficient evidence shows the exact relationship of DNMT1 with immune regulators in AML patients characterized by a poor response to chemotherapy or anti-immune therapy. Therefore, a better understanding of DNMT1 contributing to drug resistance would aid in the choice and development of strategies to treat AML.

Hypomethylation agents are based on partial incorporation into DNA, and the loss of DNMT1 is one of the markers for this process, which means that the loss of DNMT1 reflects the curative effect of decitabine to some extent [104,105]. In addition, the deficiency of normal DNMT1 function defects the response of cancer cells to decitabine, which demonstrates that DNMT1 has the potential to be a marker to predict the sensitivity of decitabine. Consistent with this hypothesis, a study performed in gliomas showed that decitabine-mediated proliferation arrest can be blocked by silencing of DNMT1, and overexpression of TERT correlated with an increase in DNMT1 levels, which improved decitabine sensitivity in the meanwhile [106]. Indeed, a low level of TERT is associated with resistance to decitabine-induced cell death in AML cell lines [92]. Thus, a similar relationship between DNMT1 and TERT or other molecules may be found in AML, which might account for the emergence of drug resistance. Recent studies have demonstrated that DNMT1 protein expression levels are positively correlated with the response to decitabine [107]. In addition, the DNMT1 level was monitored before and after applying low-dose azacytidine to predict post-transplantation AML relapse, and the result hinted that a lower DNMT1 level before treatment might indicate a better response [108]. To put it another, it is reasonable to speculate that DNMT1 may also be an important predictor of therapeutic response in AML. However, studies of this aspect in AML are relatively rare, and further studies are required to prove this.

Given the functions of DNMT1 explored before, DNMT1 has the potential to be a marker to predict drug sensitivity, it should be noted that novel therapies targeting DNMT1 could be great prospects to overcome the drug resistance.

## 6. The promise of targeting DNMT1

Accumulating evidence indicates that the expression of DNMT1 is critical to leukaemia progression, but few agents target the key regulators or axis related to the DNMT1 gene. It has been discussed previously that the interactions of DNMT1 with some crucial regulators or signalling pathways are involved in promoting leukaemogenesis. Accordingly, targeting DNMT1 is greatly promising.

Decitabine and azacytidine, two DNMTi, have shown great efficacy, although the appearance of poor response in some patients [104,109]. Mechanistically, DNMTi are mainly distinguished into nucleoside inhibitors and nonnucleoside inhibitors. The metabolite of the former, 5-aza-2′-deoxycytidine-triphosphate, is incorporated in DNA or RNA, where azacytosine replaces cytosine, and then combines with DNMT1 covalently, causing the methylation reaction that should have been activated to be blocked by azacytosine. In addition, DNA damage signalling driven by the covalent protein results in the degradation of DNMT1. While the latter interfere with the active sites of the enzymes directly, causing the function of enzymes to be arrested [89,110,111]. Recently, a DNMT1-selective inhibitor named GSK3685032 showed a powerful hypomethylation effect, which occupies the residues fall within the DNMT1 active-site loop to establish a DNMT1-DNMT1 inhibitor complex and binds DNMT1 preferentially and selectively in case the presence of hemi-methylated DNA, causing the depletion of DNMT1 and the deficiency of its function. Compared to decitabine, GSK3685032 has the ability to target DNMT1 more specifically but with less toxicity. The report also compared GSK3685032 with other small molecular DNMTi, such as GSK3510477 and GSK3484862, and achieved analogous results [112]. Despite the mounting DNMT1-targeted agents that have been produced, the problems of toxicity and tolerability *in vivo* needed to be addressed; thus, the development of therapy targeting DNMT1 is a long way. Remarkably, decitabine and all-trans retinoic acid (ATRA) inhibit DNMT1, followed by activation of miR-34a *via* promoter hypomethylation, then acquiring antineoplastic effect [113]. The studies reported before showed that miRNAs were significant during AML progression and the occurrence of drug resistance; therefore, targeting the miRNAs-DNMT1 axis will be a promising strategy to treat AML with poor response to traditional chemotherapy. Furthermore, the NCL/miR-221/NF-κB/DNMT1 axis promoting aggressive AML leukaemogenesis could be targeted by a nuclear localization signal (NLS) peptide-targeted gold nanoparticles (AuNPs) with coloaded anti-221 and AS1411 (NPsN-AS1411/a221), which could be used against AML without drug toxicity [114]. Another AuNP with adsorbed high-density lipoprotein (HDL) downregulating DNMT1 by delivering BMS309403 has been designed, which induces AML cell differentiation, and interrupts AML progression without obvious side effects [115]. Remarkably, it has been reported that bortezomib can be seen as a hypomethylation agent that induces hypomethylation by repressing the SP1/NF-kB/DNMT1 axis in AML [71]. In other words, bortezomib has the potential to target overexpressed DNMT1 occurring in AML to influence epigenetics, thus decreasing the levels of DNA methylation and increasing the expression of silenced genes. By downregulating HDAC1 and DNMT1, N-(2-aminophenyl) benzamide acridine (8a) reach the aims of anti-proliferation and promoting apoptosis, causing AML cells to be vulnerable to death. Compared to the past non-nucleoside DNMTi, 8a has shown a stronger inhibitory effect against DNMT1, which laid a foundation for its use as a drug targeting DNMT1 for the treatment of AML [116].

In fact, the functions of DNMT1 in regulating drug resistance and promoting the development of LSCs have been discussed above. We speculate that the deficiency of DNMT1 may increase the effect of chemotherapy drugs and antitumour immunity by repressing the LSCs and signalling pathways related to drug resistance to overcome the problems of drug resistance. Therefore, it is promising to improve the prognosis of relapsed or refractory AML patients by targeting DNMT1.

### 6.1. Perspectives

Although the role of DNMT1 in maintaining DNA methylation in AML is recognized widely, less attention has been given to DNMT1 than to the other epigenetic regulators in AML. Unlike DNMT3A whose mutation is often observed in AML patients and is regarded as a preleukaemic mutation promoting acute myeloid leukaemogenesis [117], DNMT1 mutation is rarely observed during the progression of AML. However, accumulating evidence indicates that overexpression of DNMT1 plays a crucial role in normal haematopoiesis and malignant blood diseases [57]. A study demonstrated that DNMT1 was correlated with a poor prognosis of human hepatocellular carcinomas. The expression level of DNMT1 increased obviously in non-differentiative cancer cells, and patients with DNMT1 positive expression had poor overall survival and relapse-free survival rates [118]. It remains unclear whether DNMT1 has the same effect in AML. Nevertheless, the functions that DNMT1 currently displays suggest that DNMT1 could be a potential predictor of sensitivity to demethylating drugs and a target in AML diagnosis and treatment.

Previously, DNMT1 was shown to play a significant role in HSCs and LSCs. A differentiation block resulting from C/EBPA mutation is a crucial step in leukaemogenesis, whereas DNMT1, as a central component of the epigenetic network is involved in this procession by regulating C/EBPA [119,120]. In contrast, a study presenting a different result demonstrated that depleting DNMT1 by decitabine before the occurrence of differentiation-inducing stimulus could maintain haematopoietic stem cell self-renewal, which is implemented by interrupting the repression of stem cell genes caused by the differentiation stimulus [121]. This is based on the temporary depletion of DNMT1 by decitabine, whereas the DNMT1 protein in normal HSCs gradually returns to normal levels. More importantly, DNMT1 as a marker to predict drug sensitivity seems feasible. In research published in 2015, DNMT1 mRNA expression was described to be strongly related to decitabine sensitivity in ovarian cancer cells, which was mediated by the RAS/MEK/DNMT1 pathway [122]. In addition to monitoring the expression of TERT and DNMT1 to predict sensitivity to decitabine in gliomas, the miRNA-DNMT1 axis plays an essential role in drug resistance and could be applied to overcome azacitidine resistance. Therefore, focussing on the importance of DNMT1 in drug resistance will provide us with a novel strategy to predict and improve the effect of AML treatment. Currently, multiple studies have proven that targeting DNMT1 directly or indirectly can improve sensitivity to chemotherapy and the prognosis of patients with high-risk factors.

Unfortunately, DNMT1 depletion by the current hypomethylating agent decitabine is transient and is S-phase dependent, which leads to an incomplete deletion of DNMT1 [123]. In addition, a study based on epigenetic therapies in patients with AML/MDS containing LSC populations showed that 5′-azacitidine and sodium valproate (VAL–AZA) therapy failed to eradicate this population [124]. Therefore, new strategies targeting DNMT1 should be explored in the future.

In conclusion, emphasizing the importance of DNMT1 in AML is indispensable in view of the few studies focussing on it. DNMT1 may be studied as an important molecule in AML accounting for its potential to be used as a potential therapeutic target to overcome therapy resistance and as a biomarker for AML prognosis and for monitoring the response to therapy.

## Author contributions

ML wrote the article; DZ revised the manuscript critically. All authors have read and agreed to the published version of the manuscript.

## Data Availability

Data sharing is not applicable to this article as no new data were created or analysed in this study.
